# Spatiotemporal dynamics of counterpropagating Airy beams

**DOI:** 10.1038/srep13463

**Published:** 2015-08-28

**Authors:** Noémi Wiersma, Nicolas Marsal, Marc Sciamanna, Delphine Wolfersberger

**Affiliations:** 1 Université de Lorraine, LMOPS/CentraleSupélec (EA 4423), Metz, 57070, France; 2 CentraleSupélec, OPTEL Research Group, LMOPS (EA 4423), Metz, 57070, France

## Abstract

We analyse theoretically the spatiotemporal dynamics of two incoherent counterpropagating Airy beams interacting in a photorefractive crystal under focusing conditions. For a large enough nonlinearity strength the interaction between the two Airy beams leads to light-induced waveguiding. The stability of the waveguide is determined by the crystal length, the nonlinearity strength and the beam’s intensities and is improved when comparing to the situation using Gaussian beams. We further identify the threshold above which the waveguide is no longer static but evolves dynamically either time-periodically or even chaotically. Above the stability threshold, each Airy-soliton moves erratically between privileged output positions that correspond to the spatial positions of the lobes of the counterpropagating Airy beam. These results suggest new ways of creating dynamically varying waveguides, optical logic gates and chaos-based computing.

## Introduction

Instabilities, self-oscillations and chaos are fundamental processes in nonlinear optics. Multiple beams’ interactions in nonlinear media, even without external feedback, can give rise to beam self-trapping and spatial solitons that may further destabilise to spatiotemporal dynamics and then, eventually, chaos^1^,^2^,^3^. Multiple parameters, such as the optical intensity or the misalignment of the interacting beams^4^,^5^, enable to control the sequence of bifurcations from stationary dynamics to deterministic chaos^6^.

Interestingly the onset of spatiotemporal instabilities observed for various beams’ configurations in different nonlinear media presents the same evolution pattern: initial diffraction, collapse to the soliton shape, then time-periodic dynamics to chaotic instabilities, where the interacting solitons rotate and twist around each other in an erratic way^7^,^8^,^9^,^10^,^11^. Solitons are self-trapped light beams propagating without change in a diffractive nonlinear medium. They are generated by the photoinduced refractive index in media, as in Kerr or photorefractive crystals or in nematic liquid crystals^10^,^12^,^13^. The interacting solitons enable various optically induced waveguiding structures in the nonlinear media. The control of the mutual exchange of energy between interacting solitons enables to create all-optical guiding, dividing and switching devices^14^,^15^ and even over large distances in different media^16^. Apart from Gaussian beams, other diffractive beam profiles such as optical vortices present solitonic behaviour under self-focusing conditions and exhibit similar dynamical routes to instabilities^17^.

Recent works have shown the possibility to induce spatial solitons from self-focusing of ideally non-diffractive beam profiles including optical Airy beams^18^,^19^,^20^. As a truncated solution of the ideal Airy waveform, the optical Airy beam has the advantage of combining the parabolic trajectory and self-healing properties of the Airy wave solution over a finite distance with the diffractive beam properties for larger propagation distances. Airy beams have been extensively studied in the recent years. First discovered in quantum mechanics^21^, the Airy wave packet has been suggested as a non-spreading and self-healing solution of the Schrödinger equation with a parabolic propagation. Due to these unique properties, the optical analogy has been of a great interest. In 2007, Christodoulides *et al.* has generated the first optical Airy beam using a spatial light modulator (SLM)^22^. The use of an SLM or other generation methods, as a photonic crystal^23^, allows for controlling the exact linear propagation trajectory of the beam^24^,^25^. Applications of the Airy beams are very large, from optical micromanipulation of particles^26^, to laser processing^27^ or optical routing^28^, including plasmonic circuitry and surface tweezers using Airy plasmons^29^.

As already mentioned, under high-focusing conditions the Airy beam undergoes soliton-like behaviour. The main part of the beam power is focused in a so-called off-shooting soliton while the remaining part propagates as in the linear case. Non-stationary dynamics of a single Airy-soliton have been shown in Kerr media and called moving solitons^30^. The collision of two Airy beams also suggests a large variety of interaction schemes. The solitonic interactions have been demonstrated in the spatial domain using co-propagating Airy beams^31^,^32^,^33^,^34^ and in the temporal domain using Airy pulses^35^,^36^. Recently we have studied the counterpropagating (CP) configuration in photorefractive media^37^. First results have shown more complex stationary waveguide structures than those induced by interacting Gaussian beams. A single Airy beam leads to waveguiding structures with multiple outputs. The additional interactions induced by a CP beam allows for achieving complex waveguiding structures that would otherwise require the CP interactions of more than two Gaussian beams. But these stationary structures only exist for a limited range of the nonlinearity strength. As will be shown here, by increasing the nonlinear coupling strength, spatiotemporal dynamics appear that result from the interactions of the two counterpropagating (CP) Airy beams and that differ from what is known from CP Gaussian beams’ systems.

In this paper we analyse numerically the spatiotemporal dynamics of two incoherent counterpropagating Airy beams interacting in a photorefractive crystal under focusing conditions. When a positive external electrical field is applied on the crystal, multiple waveguiding structures are photoinduced. If we increase the nonlinearity of the system, we demonstrate the existence of a threshold curve above which non steady-state dynamics appear. The threshold leading to spatiotemporal dynamics can be tuned by the crystal length, the nonlinearity strength and the beams’ intensities. For similar operating conditions this threshold is larger for interacting Airy beams when compared to interacting Gaussian beams, hence demonstrating the larger stability range of the photoinduced Airy waveguides. Above the threshold the position of the off-shooting soliton varies periodically in time. The period is of the same order of magnitude than the material nonlinear optics time-scale and the amplitude is determined by the Airy properties, hence both properties can be controlled by the beam interaction scheme. When further increasing the nonlinear strength and/or the crystal length, this time-periodic dynamics bifurcates to a chaotic-like dynamics of the off-shooting soliton. The erratic motion of the soliton encompasses privileged positions that match the input positions of the multiple lobes of the counterpropagating Airy beams. The engineering of the Airy beam properties therefore allows to modify the topology of the resulting chaotic motion. These findings not only address the important issue of the Airy waveguiding stability but also suggest new ways of creating dynamically varying waveguides, optical logic gates and chaos-based computing.

## Results

### Modelisation and simulation scheme of two counterpropagating Airy beams

**Figure 1:**
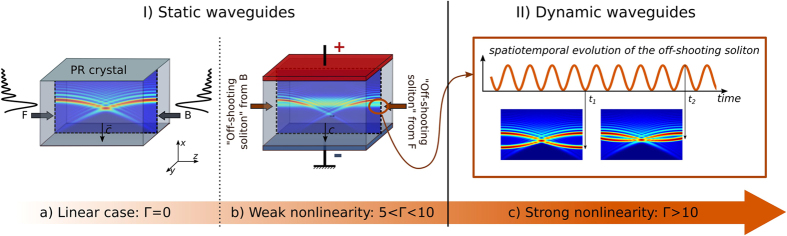
Two counterpropagating Airy beams’ configuration in a photorefractive crystal. (**a**) Linear propagation in an unbiased photorefractive crystal (Γ = 0). (**b**–**c**) Nonlinear interaction scheme of two counterpropagating Airy beams in an externally biased photorefractive crystal: (**b**) intensity field inside the crystal induced by weak nonlinear interactions, (**c**) spatiotemporal evolution of the forward “off-shooting soliton” for stronger nonlinear interactions at *z* = *L*.

To study the nonlinear interactions of two CP Airy beams in a photorefractive medium, we consider the propagation of two identical one-dimensional Airy beams along the longitudinal *z*-axis. The two Airy beams are initially injected at each side of the crystal and have both the lobe size 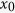 and aperture parameter *a*, as given by the following equations^8^:


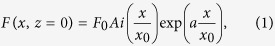



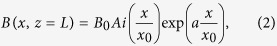


where *F*_0_ and *B*_0_ correspond to the electric field amplitudes of respectively the forward beam *F* and the backward beam *B*, *Ai* represents the Airy function, *x*_0_ an arbitrary transverse scale and *a* the truncation factor. Counterpropagating beams are herein defined by their opposite propagation directions along the longitudinal *z*-axis [Fig. 1]. Each Airy beam is formed of successive lobes, the first order (centered at *x* = −*x*_0_) being the main lobe. The nonlinear propagation of these two incoherent CP beams can be expressed as follows:


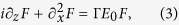



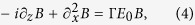


where 
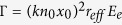
 is the nonlinear photorefractive coupling strength (*r*_*eff*_ is the effective component of the electro-optic tensor and *E*_*e*_ the external electric field), *E*_0_ is the homogeneous part of the x-component of the photorefractive space-charge field. The temporal evolution of *E*_0_ is calculated using a relaxation-type dynamics given by: 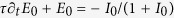, where *τ* is the relaxation time of the crystal and 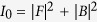. A positive external electrical field is applied along the *c*-axis of the crystal (parallel to the *x*-axis) [Fig. 1(b)]. Through the Pockels effect the optical Airy beams locally photoinduce a refractive index variation in the photorefractive crystal which leads to optically induced complex waveguiding structures in the photorefractive nonlinear material. The induced refractive index distribution is then related to the combination of the multiplexed focused Airy beams *F* and *B*.

Under focusing conditions, the Airy beam undergoes nonlinear interactions inside the photorefractive medium and most of the beam turns into an “off-shooting soliton”, while a small fraction of the power remains a self-accelerating linear packet [Fig. 1(b)]^18^,^30^. As shown recently^37^, the refractive index variation structure photoinduced by a single or two CP Airy beams enables to guide optical beams along the crystal similar to systems of two CP conventional beams^14^. As interactions of two CP Gaussian beams or two CP vortices lead to similar spatiotemporal dynamics, we question whether self-accelerating Airy beams undergo a similar spatiotemporal behaviour and how the Airy properties influence the dynamics. To describe these dynamics, we analyse the spatiotemporal evolution of the output position of the forward off-shooting soliton at the crystal’s output plane *z* = *L* [Fig. 1(c)].

### Onset of spatiotemporal dynamics

**Figure 2:**
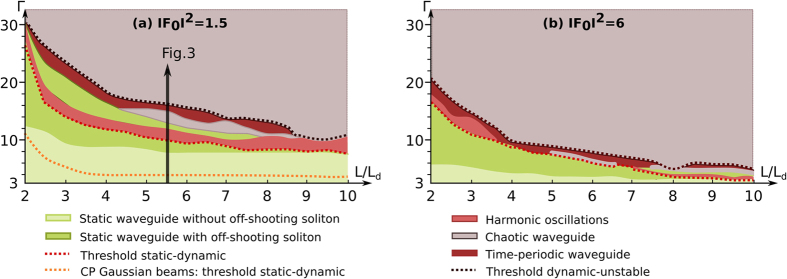
Typical dynamical behaviour of counterpropagating Airy beams in the parameter plane (Γ, *L*): (**a**) with low input intensities 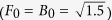, (**b**) with high input intensities 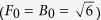.

The interaction schemes of two CP beams depend on two main control parameters, that are the nonlinear coupling constant Γ and the crystal length *L*. Figure 2 shows a stability diagram in the plane of the following parameters: (Γ, *L*). It depicts the various spatiotemporal dynamics of the forward-propagating “off-shooting” soliton’s intensity at the output *I*(*x*, *z* = *L*) for two different intensities 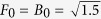 [Fig. 2(a)] and 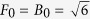 [Fig. 2(b)]. For an Airy lobe’s waist *x*_0_ = 10*μm* (*a* = 0.01), the parameter range corresponds to 1*cm* ≤ *L* ≤ 10*cm* and, for Γ, an external electrical bias field of a few *kV*/*cm*.

**Figure 3:**
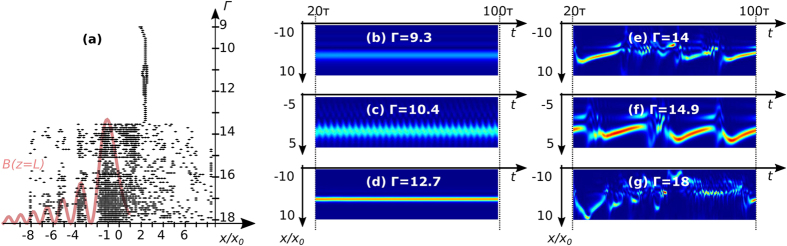
Spatiotemporal dynamics of two counterpropagating Airy beams in a long crystal *L* =  **5.5*****L***_***d***_**, with the normalized intensities** 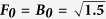. (**a**) Bifurcation diagram of the transverse output position of the forward off-shooting soliton at *z* = *L*, with the transverse normalized intensity profile of backward Airy beam at *z* = *L*. (**b–g**) Temporal evolution of the transverse output position of the forward off-shooting soliton at *z* = *L*: (**b**) steady-state (Γ = 9.3), (**c**) sinusoidal oscillations (Γ = 10.4), (**d**) second steady-state (Γ = 12.7), (**e**) first instabilities (Γ = 14), (**f**) periodical non-sinusoidal oscillations (Γ = 14.9) and (**g**) instabilities (Γ = 18). E.g. experimentally for CP Airy beams in a SBN:75 crystal (L_*_5mm_*_5*mm*) with *x*_0_ = 10*μm*: *L* = 28*mm*, *U*_*ext*_ ∈ [500*V*, 900*V*].

For low Γ-values (Γ = 3), the nonlinearity Γ applied on the system is not high enough to create locally a large refractive index variation inside the crystal by the photorefractive effect and therefore to induce an off-shooting soliton. Still, the propagation of each Airy beam optically induces a curved waveguide along the deflecting Airy trajectory^28^. We call this region ‘static waveguide without off-shooting soliton’. For a larger nonlinearity strength, each CP Airy beam undergoes self-trapping and a part of the beam’s energy turns into an “off-shooting” soliton [Fig. 1(b)]. We define the existence of an off-shooting soliton, when at least 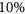 of the input intensity exits at *z* = *L* and can be clearly distinguished from the linear output beam. Since almost half of the energy is stored in the first Airy lobe^30^, the nonlinearity of the system mostly influences the main lobes and the off-shooting solitons. The interaction of the two CP Airy beams then leads to various new static waveguide structures and we call this region ‘static waveguide with off-shooting soliton’. As presented in reference^37^, the photoinduced waveguide structure enables a Gaussian beam to exit the crystal at a single or at two output positions simultaneously. The parabolic trajectory of the CP Airy beams enables waveguiding structures even for transverse shifts of the interacting beams that by far exceed the beam waist. When we still increase the nonlinearity Γ, the waveguide is no longer steady in time but rather shows stable time-periodic dynamics: the off-shooting soliton evolves from a constant transverse output position to an output position that oscillates harmonically in time along the *x*-axis [Figs 1(c) and 3(c)]. We call this region ‘harmonic oscillations’. Similar to the case of CP Gaussian beams^8^, the critical nonlinearity strength that delimits the onset of time-periodic oscillations of the waveguide decreases with the increase of the crystal length *L*, see the line labelled ‘threshold static-dynamic’ in Fig. 2.

For an even larger Γ and/or crystal length *L*, the time-periodic waveguide dynamics is replaced by chaotic-like spatiotemporal dynamics. The position of the off-shooting soliton does not vary periodically in time but rather in an erratic way. As will be shown later, while the trajectory is erratic in time, the motion of the off-shooting soliton is attracted towards the input positions of the lobes of the counterpropagating Airy beam. We call this parameter region ‘chaotic waveguide’. The critical nonlinearity leading to unstable waveguiding decreases with the increase of the crystal length *L*, as is also true for CP Gaussian^8^ and vortex beams (see the line labelled ‘threshold dynamic-unstable’ in Fig. 2).

Interestingly, we identify two additional regions. In both cases (a) and (b) the time-periodic dynamically varying waveguide may re-stabilise to a static waveguide when increasing the nonlinearity. The off-shooting soliton stabilises again at a constant output position. The possibility to stabilise again the photoinduced waveguiding by increasing the nonlinearity strength has not been observed earlier with CP Gaussian beams and is related to the multilobe shape of the Airy beams. Therefore this suggests an advantage in using CP Airy beams. We also identify another parameter region where the position of the off-shooting soliton varies periodically in time but not in an harmonic way. We have simply called this region ‘time-periodic waveguide’. This specific dynamics bifurcates from the harmonic waveguide case but is also observed as a bifurcation of the chaotic waveguide case. We shall detail these dynamics and their bifurcations in the next section.

Similarly to other CP beams’ systems, the intensity of the input beams is an important parameter. When increasing the total optical intensity injected in the crystal through the CP beams the refractive index variations increases, hence resulting in more nonlinear interactions; see [Fig. 2(b)]. When we compare the Fig. 2(a,b), the critical nonlinearity that leads to either a time-periodic waveguide or even chaotic waveguide for a normalized intensity 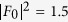 [Fig. 2(a)] is larger than for 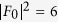 [Fig. 2(b)]. The stability of the waveguide is therefore reduced by the increase of the optical intensity.

Finally it is worth comparing the critical nonlinearity that leads to dynamically varying waveguide (our dashed line) in the case of CP Airy beams with the one computed for CP Gaussian beams (dotted line). Besides the fact that Airy-induced waveguides have more complex features than Gaussian-induced waveguides, it appears also that, the Airy-induced waveguides are stable in a large range of parameters and in particular for a large range of nonlinearity strength and/or crystal length. This unique property of Airy-induced waveguides is related to the diffraction-free propagation and multilobe shape of Airy beams.

### Detailed route to chaos

In this section we analyse the nature and the evolution of the spatiotemporal dynamics of two CP Airy beams for a fixed crystal length *L* when the nonlinear coupling strength Γ is increased. Physically the nonlinearity is increased through the positive electrical bias field applied on the crystal. Although as mentioned earlier the stability of the photoinduced waveguide depends on both the crystal length *L* and the beam intensities, we shall restrict ourselves to one case where 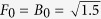 and *L* = 5.5*L*_*d*_. For Airy beams with the parameters *x*_0_ = 10*μm*, *a* = 0.1, it corresponds to a crystal length of *L* = 28*mm* (see arrow [Fig. 2(a)]). This case illustrates the complexity underlying the sequence of bifurcations to spatiotemporal instabilities of the waveguide [Fig. 3(a)]. A similar sequence of bifurcations occurs when varying the system parameters. For each 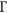-value, we simulate the propagation of two CP Airy beams over *t*_*f*_ = 100*τ*_0_, where at each crystal’s side the main lobe of the CP Airy beams is centered around *x* = −*x*_0_ for its input position. We then display the spatiotemporal dynamics of the forward off-shooting soliton at the crystal’s output side *z* = *L* along the transverse *x*-axis by plotting the off-shooting transverse position versus time [Fig. 3(b–g)]. To avoid the transient dynamics, we detect the extreme 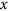-positions taken by the off-shooting soliton within the times *t*_1_ = 20*τ*_0_ and *t*_*f*_ = 100*τ*_0_. The bifurcation diagram on Fig. 3(a) resumes the position of the spatial output of the off-shooting soliton during time: for each 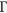-value, the various dots display the *x*-extrema taken by the off-shooting soliton along time.

**Figure 4:**
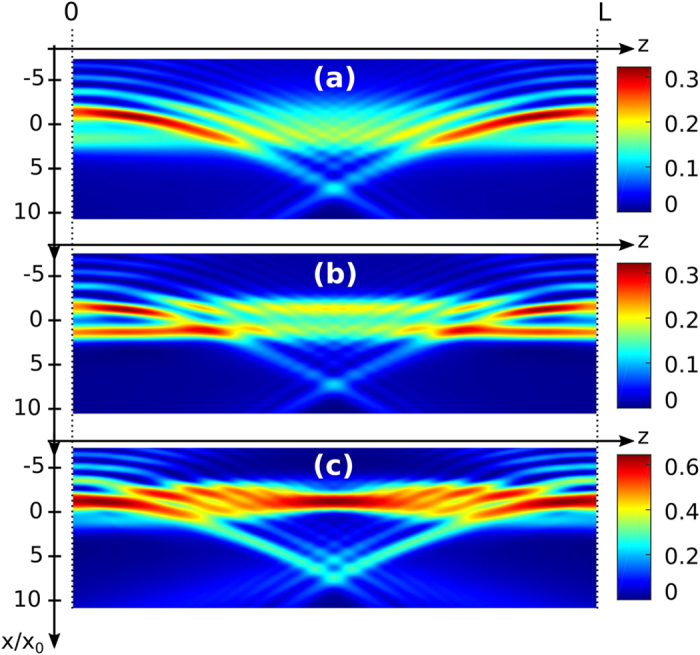
Static waveguides: intensity fields of two CP Airy beams inside a PR crystal under focusing conditions (*L* = **5.5*****L***_***d***_). With initial intensity 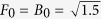 [Fig. 2(a)]: (**a**) below the threshold curve (Γ = 10), (**b**) re-stabilisation above the threshold curve (Γ = 12.5). (**c**) With initial intensity 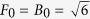, on the threshold curve (Γ = 7) [Fig. 2(b)].

The diagram on Fig. 3(a) displays the route to instabilities from a system with a weak nonlinearity (Γ = 9) to a highly nonlinear system (Γ > 16). For 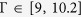, the bifurcation diagram displays a steady-state transverse output position of the off-shooting soliton during time [Fig. 3(b)]. The steady-state case depicted in Fig. 3(b) corresponds to the waveguide structures demonstrated in^37^, where the CP Airy beams and their off-shooting solitons co-exist in the crystal. When 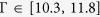, two extrema of the *x* position of the off-shooting soliton appear for a given Γ value. The time-trace of the 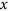 position of the off-shooting soliton displays a sinusoidal evolution [Fig. 3(c)]. We observe a stable oscillating dynamics, where the off-shooting soliton rotates periodically around its characteristic position *x* = 2.5*x*_0_. The period of the sinusoidal oscillation is about 3.5*τ*_0_, i.e. is of the same order of magnitude than the material nonlinear optics time-scale. The amplitude of the oscillation is determined by the Airy properties and in particular their deflection characteristics. Indeed the amplitude of the oscillation is larger for a longer crystal since by increasing the crystal length, the CP Airy beams deflect more before colliding. The Fig. 3(d) shows that this oscillating soliton dynamics re-stabilises when increasing the nonlinearity leading to a new static waveguide structure. This singular case will be explained in details and illustrated in Fig. 4(b) of the next section.

When increasing the nonlinearity strength above Γ = 13, the position of the off-shooting soliton presents an erratic motion along the output plane [Fig. 3(a)]. Figure 3(e) indicates that the soliton tends to follow alternatively a complex time-periodic, then a chaotic-like behaviour. As depicted on Fig. 3(f), when we increase the nonlinearity (Γ = 14.8), the chaotic-like evolution of the position of the off-shooting solution stabilises to a time-periodic dynamics where oscillations at a slower time-scale modulate the dynamics with a higher amplitude than for the harmonic oscillation depicted in Fig. 3(c).

Finally when Γ > 15.3, the time-periodic dynamically varying waveguide becomes unstable and the position of the off-shooting soliton rotates in an erratic way around the single Airy case position *x* = 0 and the CP main lobe’s position *x* = −*x*_0_ [Fig. 3(g)]. It is worth noting that for a very high coupling strength (Γ > 16.3) the erratic motion of the off-shooting soliton encompasses additional attractive *x*-values at 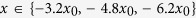. Interestingly these *x*-values correspond to the respective input positions of the second, third and fourth lobe orders of the CP Airy beam [Fig. 3(a)]. The characteristics of this chaotic soliton motion will be further discussed in the next sections.

### Re-stabilisation of the waveguide for large nonlinearity strength

As previously emphasized in Fig. 2, dynamically varying waveguides photoinduced by two counterpropagating Airy beams can re-stabilise when the nonlinearity Γ increases. So far, the dynamical behaviour of two CP Gaussian beams has only shown an evolution from a steady-state, then time-periodic to chaotic like regimes. The Fig. 4 compares the typical static waveguides with off-shooting soliton that can be observed in the two stability diagrams [Fig. 2(a–b)]. Figure 4(a) corresponds to the waveguide structures presented in^37^ where a Gaussian probe beam can be guided along the crystal to one or two outputs. Figure 4(b) illustrates the new waveguide structure in the re-stabilization zone (‘static waveguide with off shooting soliton’ above the line labelled ‘threshold static-dynamic’ [Fig. 2]). This waveguide structure offers the same type of photoinduced waveguides as in Fig. 4(a) but with a better coupling efficiency (up to 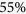 instead of 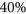) in the off-shooting solitons due to the stronger focusing nonlinearity. Figure 4(c) also presents a particular steady-state structure in the strong intensity case 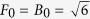 at two parameter points: (*L* = 5, Γ = 7.5) and (*L* = 5.5, Γ = 7). Contrary to the usual steady-state case, where the CP Airy beam induces a transverse shift of the offshooting soliton^37^, the off-shooting soliton changes its output position and merges exactly into the main lobe of the CP Airy beam. In the configurations (b) and (c) where the nonlinearity of the system is increased through 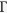 or the initial intensity, the space-charge field photoinduced by the multiple lobe orders of the two CP Airy beams has a significant role in the interaction schemes in the photorefractive crystal. Although the main power is transferred into the off-shooting soliton during the nonlinear propagation of the Airy beams^30^, the secondary lobes of the Airy beams are essential for the re-stabilization of our system above the conventional steady-state threshold curve.

### Chaotic motion of Airy-induced soliton

As previously emphasized, the dynamical behaviour of the photoinduced waveguide significantly depends on the crystal length. In the ‘chaotic waveguide’ region [Fig. 2], we propose therefore to compare the situation of a short crystal (e.g. *L* = 2.5*L*_*d*_ = 13*mm*) and the one of a long crystal (e.g. *L* = 5.5*Ld* = 28*mm*). It is worth mentioning that due to their parabolic trajectory, the two CP Airy beams intersect at 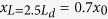 for the short crystal and at 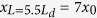 for the long crystal. As a result, the photoinduced waveguides originate mostly from the interaction of the first and second lobe orders of the CP Airy beams in the case of a short crystal, and from the interaction of the four first lobe orders of the CP Airy beam in the case of a longer crystal. The resulting waveguiding structure in the case of a longer crystal will be larger along the transverse 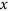-axis (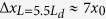 instead of 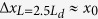 in the case of a short crystal). Similarly, in the case of a long crystal, the transverse trajectory of the off-shooting solitons will shift from its typical transverse position *x*_*soliton*_ = 0 towards the + *x*-direction.

**Figure 5:**
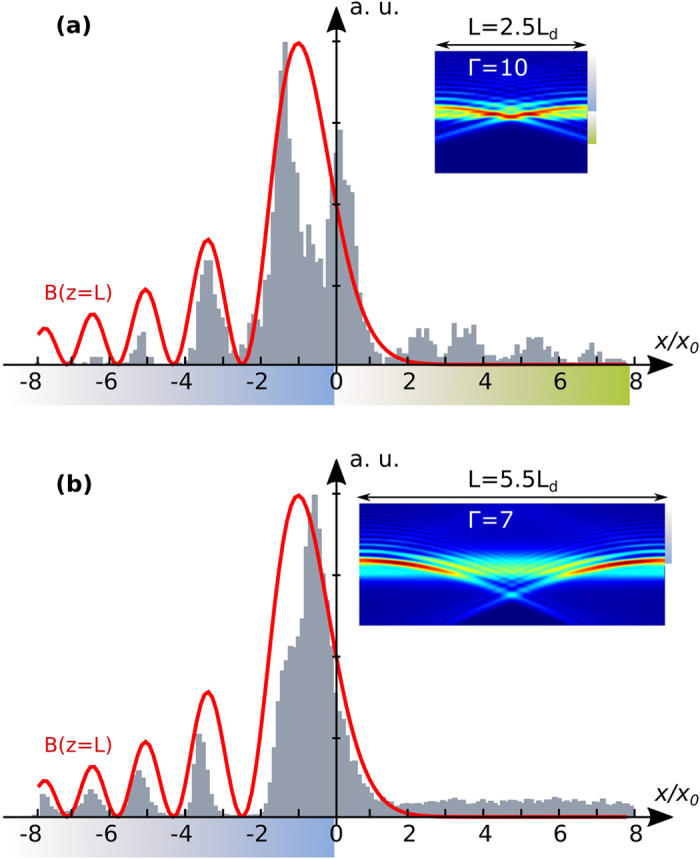
Probability of occurrence of the position of the off-shooting soliton at the output face of (**a**) a short crystal (*L* = 2.5*L*_*d*_; Γ > 26.6) and (**b**) a long crystal (*L* = 5.5*L*_*d*_; Γ > 15.3), with 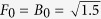 in both cases. The red curve indicates as a guide for the eyes the position of the counterpropagating Airy beam at the crystal output face. Insets represent the photoinduced waveguide in a situation before the onset of spatiotemporal dynamics.

In particular, as illustrated in Fig. 3(a,g) for a long crystal, under high nonlinear conditions, the system of two CP Airy beams shows a peculiar instability pattern: the output positions of the off-shooting soliton in the unstable regime appear to be attracted toward very specific output positions, which correspond to the respective input positions of the different lobe orders of the CP Airy beam. Figure 5 depicts the statistical distribution of the output position of the off-shooting soliton in (a) a short crystal (*L* = 2.5*L*_*d*_) and (b) in a long crystal (*L* = 5.5*L*_*d*_) with 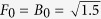. The output positions of the off-shooting soliton are not distributed in a continuous way but rather in a discrete way. The privileged output positions match with the input positions of the Airy lobes of the CP backward beam (blue zone). Also the attraction strength, measured by the highest probability in the plotted histograms, decreases for the higher lobe orders, as the space-charge field related to the energy distribution of the Airy beam decreases along the −*x*-axis. In the short crystal case (a), the off-shooting soliton is also attracted towards the + *x*-axis, at the output positions of the Airy lobes of the forward beam (green zone).

Such spatially localized instabilities have never been observed in an optical system using CP beams^8^,^11^. Our system made of CP Airy beams therefore creates a chaotic motion of the off-shooting soliton whose topology can be engineered by both the Airy beam properties and the photorefractive crystal nonlinearity strength and length. Recent years have seen a tremendous interest in applications of optical chaos for all-optical signal processing including optical generation of random numbers. The most conclusive proposals so far have used the temporal chaotic output of semiconductor lasers^38^. The digital sampling of optical chaos allows to extract random bits at high bit rate^39^. The extension to massive parallel computing is however limited in that it requires either a large number of such chaotic lasers or the use of uncorrelated emission from individual laser longitudinal or transverse modes. In the present scheme, one has access to a chaotic output (the erratic motion of the off-shooting soliton) that is by essence spatially multiplexed at discrete positions that match the locations of Airy beam lobes. Our findings therefore suggest innovative ways of performing multiplexed chaos-based optical computing.

## Discussion

To conclude, the interaction of two CP Airy beams in a photorefractive crystal leads to peculiar spatiotemporal dynamics. The system evolves from static to time-periodic then chaotic waveguides when increasing the nonlinearity strength and the crystal length. We demonstrated the existence of a threshold curve above which non steady-state dynamics appear. By comparison to similar studies using CP Gaussian beams, photoinduced Airy waveguides are stable for a larger range of parameters. Also on the route to instabilities, we identify a singular additional region where dynamical waveguides re-stabilise to static waveguides with a better coupling efficiency. When the system bifurcates to the chaotic-like dynamics, the off-shooting soliton moves in an erratic way with privileged positions that match the input positions of the multiple lobes of the CP Airy beams. Such spatially localized instabilities suggest innovative ways of performing optical computing based on spatiotemporal chaos. The unique properties of static and dynamic Airy waveguide structures motivate experimental demonstration and implementation in different nonlinear optical media.

## Additional Information

**How to cite this article**: Wiersma, N. *et al.* Spatiotemporal dynamics of counterpropagating Airy beams. *Sci. Rep.* **5**, 13463; doi: 10.1038/srep13463 (2015).
